# Methanolic Extract of the Herb *Ononis spinosa* L. Is an Antifungal Agent with no Cytotoxicity to Primary Human Cells

**DOI:** 10.3390/ph13040078

**Published:** 2020-04-24

**Authors:** Dejan Stojković, Maria Inês Dias, Danijela Drakulić, Lillian Barros, Milena Stevanović, Isabel C. F. R. Ferreira, Marina D. Soković

**Affiliations:** 1 Department of Plant Physiology, Institute for Biological Research “Siniša Stanković”- National Institute of Republic of Serbia, University of Belgrade, Bulevar despota Stefana 142, 11000 Belgrade, Serbia; dejanbio@ibiss.bg.ac.rs; 2 Centro de Investigação de Montanha (CIMO), Instituto Politécnico de Bragança, Campus de Santa Apolónia, 5300-253 Bragança, Portugal; maria.ines@ipb.pt (M.I.D.); iferreira@ipb.pt (I.C.F.R.F.); 3 Institute of Molecular Genetics and Genetic Engineering, University of Belgrade, Vojvode Stepe 444a, 11042 Belgrade, Serbia; danijeladrakulic@imgge.bg.ac.rs (D.D.); milenastevanovic@imgge.bg.ac.rs (M.S.); 4 Faculty of Biology, University of Belgrade, Studentski trg 16, 11000 Belgrade, Serbia; 5 Serbian Academy of Sciences and Arts, Knez Mihailova 35, 11001 Belgrade, Serbia

**Keywords:** *O. spinosa*, methanolic extract, aerial parts, phenolic compounds, antifungal, antibiofilm, cytotoxicity

## Abstract

*Ononis spinosa* L. is a plant traditionally used as folk remedy. There are numerous studies regarding chemical constituents and health beneficial properties of Ononidis Radix. The following study was designed to investigate chemical composition and antifungal potential of the methanolic extract obtained from the *O. spinosa* L. herb. Chemical analyses regarding phenolic compounds of *O. spinosa* were performed by liquid chromatography with mass spectrometry (LC-DAD-ESI/MSn). Antifungal activity, antibiofilm properties and antifungal mode of action of the extract were evaluated, as well as cytotoxicity. Chemical analyses revealed the presence of flavonoids, isoflavonoids and phenolic acids in *O. spinosa,* with kaempherol-*O*-hexoside-pentoside being the most abundant compound (5.1 mg/g extract). Methanolic extract was active against all of the tested microfungi with *Penicillium aurantiogriseum* being the most sensitive to the extract inhibitory effect at 0.02 mg/mL; and effectively inhibited biofilms formed by *Candida* strains. Minimum fungicidal concentrations of extract rose in the presence of ergosterol and leakage of cellular components was detected. The extract showed no cytotoxicity to human gingival fibroblast (HGF-1) cells. This study significantly contributes to overall knowledge about medicinal potential of *O. spinosa* herbal extract and enlightens previously unrevealed properties. *O. spinosa* aerial parts seem to be an interesting candidate for the development of antifungal preparations, non-toxic to human cells.

## 1. Introduction

Colossal structural diversity and biological activity of natural molecules are unrivaled by any available synthetic drugs in reference libraries. As such, these privileged platforms derived from nature serve as important scaffolds for the design of novel therapeutic candidates, including antifungals.

More than a billion people are suffering from various fungal infections, with more than 1.5 million having fatal consequences [1]. These infections are difficult to treat making the mortality rates high even in the 21th century, despite various antifungals that are currently available [1]. Even more, fungal biofilms is difficult to treat since they show resistance to the host immune system and conventional antifungal treatment when compared to free floating cells [2].

*Ononis spinosa* L. (Fabaceae), spiny restharrow an ethomedicinal plant [3], is widespread in almost all of Europe, western Asia and northern Africa [4,5]. European Pharmacopoeia has recorded *O. spinosa* dried roots as “Ononidis Radix”. The roots of *O. spinosa* are known to be used as diuretic and anti-inflammatory agent, as well as in a variety of skin conditions, including itches, wounds, burns and dermatitis (eczema) [6,7,8,9]. The ashes obtained after burning the plant samples of *O. spinosa* were previously shown as active against different *Candida* species [3]. Previous phytochemical investigation of *O. spinosa* revealed the presence of flavonoid derivatives, sterols and terpene compounds [10,11,12]. Previous antifungal investigations of plants belonging to *Ononis* genera have shown that extracts obtained from *Ononis* species have antibacterial and antifungal effects [13,14].

Bearing in mind that most of the investigations of *O. spinosa* as healing plant are focused on the analysis of the phytochemicals and health beneficial properties from the roots, and that ethno-medicinal use of *O. spinosa* aerial parts is limitedly described in the literature, the aim of this study was to identify phenolic compounds presented in aerial parts of *O. spinosa* and to investigate potential antifungal properties of methanolic extract against wide spectrum of fungal pathogens and contaminants. Additionally, effects of the methanolic extract on the biofilm formation by *Candida albicans*, *C. tropicalis* and *C. krusei* have been analyzed. Furthermore, analysis of ergosterol and cytoplasmic membrane as potential targets for the activity of the extract was set as one of the goals of this study, as well as the cytotoxic effect of methanolic extract on primary human gingival fibroblast cells.

## 2. Results and Discussion

### 2.1. Chemical Composition of Phenolic Compounds

**Figure 1:**
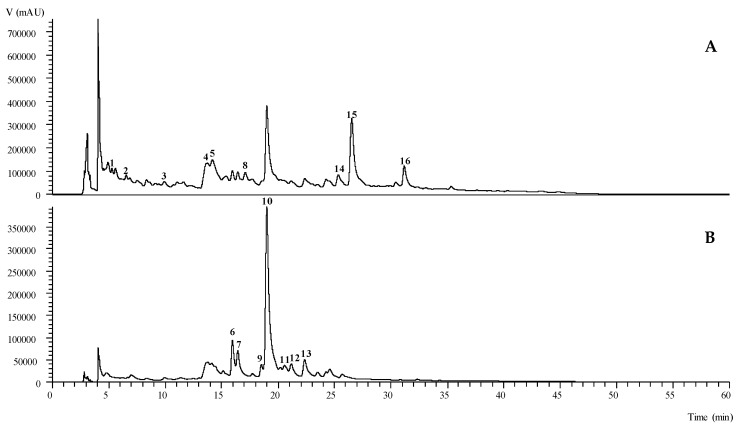
Retention time (Rt), wavelengths of maximum absorption in the visible region (λ_max_), mass spectral data and tentative identification of the phenolic compounds present in *Ononis spinosa* L., recorded at 280 nm (**A**) and 370 nm (**B**).

The chromatographic data obtained from the High-Performance Liquid Chromatography coupled with a Diode Array Detector and Electrospray Mass Spectrometry (HPLC-DAD-ESI/MSn) analyses of the phenolic compounds in the extracts of *O. spinosa* are presented in the Table 1 and Figure 1. We have identified 16 compounds in the extract, which are counting seven flavonoids, five phenolic acids and four isoflavonoids. Chromatographic characteristics corresponding to standard compounds caffeic acid, quercetin-3-*O*-glucoside and kaempherol-3-*O*-glucoside, were used for positive identification of Peaks **3**, **9** and **13**, respectively. The most abundant class of the compounds were flavonoids with the highest number of tentatively identified compounds and with the highest quantity (12.2 ± 0.1 mg/g extract) as well. Peaks **7** ([M − H]^−^ at *m*/*z* 609), **10** ([M − H]^−^ at *m*/*z* 579) and **12** ([M − H]^−^ at *m*/*z* 447), had given a particular MS^2^ fragment at *m*/*z* 285 (kaempherol aglycone), analogous to the loss of 324 u (two hexosyl units), 294 u (one hexosyl and one pentosyl unit) and 162 u (one hexosyl unit), respectively. These Peaks (**7**, **10** and **12**) were tentatively identified as kaempherol-*O*-dihexoside, kaempherol-*O*-hexoside-pentoside and kaempherol-*O*-hexoside (with a different retention time when compared to the peak **13**), respectively. According to their chromatographic characteristics, Peaks **6** and **11** were found to be glycosylated derivatives of quercetin, and these were further identified as quercetin-*O*-hexoside-pentoside and acetylquercetin-*O*-hexoside, respectively. As far as the authors knowledge there are no previous reports on *O. spinosa* regarding the identification of this type of flavonoids. Nevertheless, these types of compounds have been previously identified in others *Ononis* varieties, such as *O. arvensis* [15] and *O. angustissima* L. [16] aerial parts.

Regarding the phenolic acids group, peaks **1** ([M − H]^−^ at *m*/*z* 341) and **2** ([M − H]^−^ at *m*/*z* 355) were tentatively identified as caffeic acid hexoside and ferulic acid hexoside, respectively, based on its characteristic Ultraviolet–Visible (UV-Vis) spectra, fragmentation pattern and the information previously reported by Barros et al., [17]. Peaks **4** and **5** ([M − H]^−^ at *m*/*z* 473) were tentatively identified as the *cis* and *trans* isomers of chicoric acid, respectively, based on the chromatographic information previously reported by Chen et al., [18]. As previously mentioned, to the best of our knowledge there are no studies, on the composition in phenolic acids in *O. spinosa*. Nevertheless, these types of compounds have already been reported in the *O. angustissima* L. aerial parts [16].

Finally, the groups of isoflavonoids found in *O. spinosa* were less abundant in comparison to the other two groups of phenolic compounds. Though, this group has been extensively studied in *O. spinosa* [19,20,21,22]. Peaks **8** ([M − H]^−^ at *m*/*z* 459), **14** ([M − H]^−^ at *m*/*z* 489), **15** ([M − H]^−^ at *m*/*z* 459) and **16** ([M − H]^−^ at *m*/*z* 515), were tentatively identified as spinonin-*O*-hexoside, pseudobaptigenin-*O*-hexoside, formononetin derivative and formononetin-*O*-malonyl-hexoside, respectively, based on its chromatographic characteristic, as also their fragmentation pattern, which has been previously reported by Gampe et al. [5]. Although two of these compounds were found in trace amounts in the studied sample (peaks **8** and **16**), it is important to highlight the relevance of isoflavonoids to human health, having already been intensively studied, mainly in legumes, for their effects to inhibit the proliferation of certain types of cancers or even against some neurodegenerative diseases [23,24].

### 2.2. Antifungal Activity of O. spinosa Methanolic Extract

Antifungal activity of the methanolic extract obtained from the aerial parts of *O. spinosa* is presented in Table 2. The activity of extract was tested against wide range of pathogenic and contaminant fungi, including human, animal and plant pathogens, as well as food contaminant species.

Antifungal activity of *O. spinosa* was the most prominent against food isolated species *Penicillium aurantiogriseum* with minimum inhibitory concentration (MIC) of 0.02 mg/mL and minimum fungicidal concentration (MFC) of 0.04 mg/mL. On the other hand, the most resistant species to the effect of *O. spinosa* methanolic extract was *Penicillium ochrochloron*, a species frequently isolated from the soil and apples, with MIC of 5.00 mg/mL and MFC of 10 mg/mL. Antifungal activity of tested extract was the most prominent against *Penicillium aurantiogriseum* followed by *Aspergillus fumigatus*, *Candida tropicalis*, *A. versicolor*, *A. niger*, *Trichoderma viride*, *P. funiculosum*, *C. albicans*, *C. krusei*, *A. ochraceus* and *P. ochrochloron*. As far as we know, this is the first study reporting antifungal activity of the methanolic extract obtained from the herb of *O. spinosa*.

The activity of *O. spinosa* was comparable to the activity of commercial fungicides. The most promising effect was achieved on *A. fumigatus* and *P. aurantiogriseum*, to which commercial antifungal drugs ketoconazole and bifonazole showed weaker activity when compared to the antifungal action of *O. spinosa*. Most of the tested microfungi strains gave the similar results regarding MICs and MFCs, which were in the activity range of tested commercial positive controls (ketoconazole and bifonazole).

Previous literature data indicated antifungal potential of extract obtained from the roots of *O. spinosa*, which is traditionally used in ethnomedicine [25]. Results obtained in this study indicate that *O. spinosa* methanolic extract obtained from the aerial plant parts possessed antifungal properties as well. A study by Deliorman Orhan et al., [25] indicated that the infusion made from Ononidis Radix is active against the following fungal species: *Candida albicans* (MIC 0.016 mg/mL; MFC 0.064 mg/mL), *C. tropicalis* (MIC 0.016 mg/mL; MFC 0.064 mg/mL), *C. parapsilopsis* (MIC 0.008 mg/mL; MFC 0.016 mg/mL), *Trichophyton rubrum* (MIC 0.016 mg/mL; MFC not active), *Epidermophyton floccosum* (MIC 0.066 mg/mL; MFC not active) and *Microsporum gypseum* (MIC 0.032 mg/mL; MFC not active) [25]. Although in the paper by Deliorman Orhan et al., [25] the antifungal action of the *O. spinosa* root infusion was analyzed, obtained results are comparable to ours. Furthermore, ethanolic and water solutions of the ashes obtained from *O. spinosa* plant [3] showed anticandidal activity. Fungicidal concentrations were in range of 1.25 µg/mL (towards *C. albicans*) to 40 µg/mL (towards *C. glabrata*) for ethanolic ash solution; and from 1.25 µg/mL for *C. albicans* and to not active against *C. glabrata* for aqueous ash solution [3].

Currently, some synthetic antifungals are associated with some adverse effects and there is still no effective cure for some fungal infections in humans [26]. Namely, it has been revealed that infections caused by anthropophilic and zoophilic fungi, which represent the most common fungal infections limited to human and animal skin, nails and mucous membranes, are frequently difficult to treat with topical therapeutics and in some cases they may require long term treatment with systemic antifungals [27]. Furthermore, fungicides used in agricultural industry to prevent growth of phytopathogenic fungi may have harmful effects on the environment, as well as on humans and animals through further food chain [28]. Results obtained in this study showed that aerial parts of *O. spinosa* might provide a good basis for development of natural antifungals. Therefore, this study presents one of the many attempts to resolve issues arisen from the use of synthetic antifungal preparations, both in the treatment of humans and animals, as well as in application in agricultural industry.

### 2.3. Antibiofilm Activity of O. spinosa

In this study antibiofilm activity of the *O. spinosa* methanolic extract was tested on *C. albicans*, *C. krusei* and *C. tropicalis* (Table 3). These species are able to form structured communities that are attached to surfaces by specific signaling molecules [26]. The results of this study pointed to antibiofilm activity of *O. spinosa* (Table 3). As far as we know, these are the first results on antibiofilm activity of *O. spinosa* extract obtained from the plant aerial parts. Furthermore, MIC and MFC values of *O. spinosa* extract towards biofilms were higher when compared to MIC and MFC values obtained for free floating cells tested in microdilution assay (Table 2). *C. krusei* biofilm was more susceptible to the antibiofilm activity of *O. spinosa*. Importantly, the obtained results for *O. spinosa* extract (MIC 2.5 mg/mL and MFC 5 mg/mL) towards biofilm of *C. krusei* are comparable to those obtained for commercial antifungal drug fluconazole (MIC 2 mg/mL and MFC 3 mg/mL), indicating the similar range of antibiofilm concentrations. Furthermore, MIC value of *O. spinosa* extract (5.00 mg/mL) obtained on *C. albicans* biofilm was lower in comparison to the MIC value of fluconazole (8 mg/mL). These results indicate very promising antibiofilm action of *O. spinosa*.

### 2.4. Insights into the Modes of Antifungal Action

**Figure 2:**
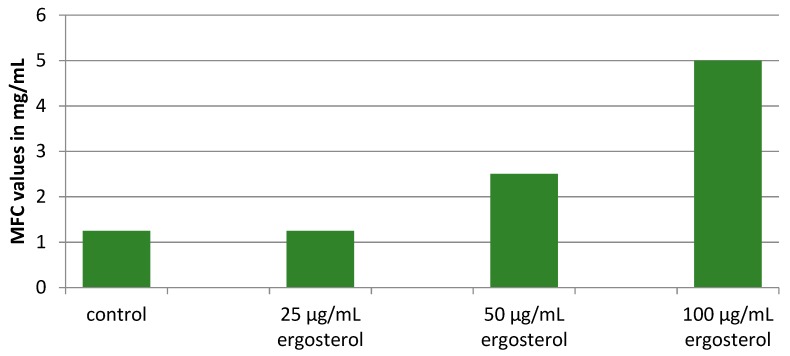
Minimum fungicidal concentrations (MFC) of *O. spinosa* methanolic extract against *C. albicans* in the presence of different ergosterol concentrations. There was no statistically significant difference between control sample and sample with added 25 µg/mL of ergosterol, while there was spastically significant difference between control ^a^, 25 ^a^ µg/mL and 50 ^b^ µg/mL and 100 ^c^ µg/mL of added ergosterol (*p* < 0.05), ^a, b, c^ statistically significant difference between samples.

Ergosterol is one of the crucial molecules found in fungal cell membranes. Since it is a vital molecule for fungal survival, the enzymes involved in its biosynthesis often present targets for the activity of effective antifungals [29]. Herein, we studied the survival of *C. albicans* in the presence of externally added increasing concentrations of ergosterol (25–100 µg/mL) and serial dilutions of the *O. spinosa* extract in order to determine whether the extract achieves its antifungal effect via disruption of ergosterol biosynthetic pathway. The results presented in the Figure 2 revealed that MFC values were increased with increasing concentrations of external ergosterol. This is leading to the conclusion that ergosterol biosynthetic pathway is disrupted by compounds presented in *O. spinosa* extract.

**Figure 3:**
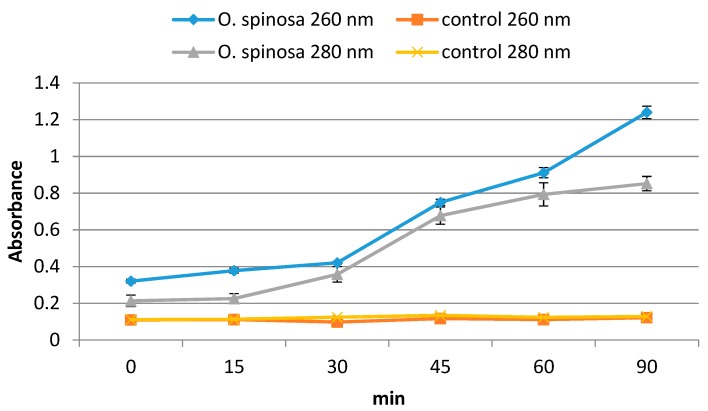
Leakage of cellular components recorded by absorbance at 260 and 280 nm in *C. albicans* treated with *O. spinosa* methanolic extract at 2 × MIC (minimum inhibitory concentration). There has been significant difference between control and treated samples (*p* < 0.05).

The effect of *O. spinosa* extract on *C. albicans* was further analyzed at the level of cell membrane permeability. A membrane permeability assay was performed in order to evaluate if breakdown of cytoplasmic membrane occurs in the presence of 2 × MIC concentration of the extract. Obtained results revealed time-dependent effect of *O. spinosa* extract on the cell membrane permeability (Figure 3). Namely, optical densities at 260 nm and 280 nm were increased rapidly after 30 min of incubation and achieved maximum values after 90 min, indicating release of intracellular components from the cells of *C. albicans* to the extracellular compartment. The results pointed out that the fungal cell membrane represents one of the targets of *O. spinosa* antifungal action.

In general, results obtained in this study presented preliminary insight into the mode of antifungal action of *O. spinosa* extract. Based on the results it could be concluded that the extract exerted its antifungal activity by disruption of ergosterol biosynthesis and by modulation of cell membrane permeability. This study represents one of the first reports exploring possible modes of action of *O. spinosa* methanolic extract obtained from the aerial parts of the plant.

Fungal pathogens have the eukaryotic conserved signaling pathways, which enable them to adapt and survive in the environment, including host cells [29]. The slight differences of fungal eukaryotic structure in relation to human cells are attractive for antifungal drug development [30]. The most important targets of antifungal drugs currently used are enzymes involved in ergosterol biosynthetic pathway [29]. Based on the literature data it might be concluded that targeting ergosterol biosynthetic pathway is the most common mode of action of major antifungals. Having in mind that ergosterol pathway is already successfully been targeted by antifungal substances currently in use [29], natural products that are found to act against targets within ergosterol biosynthetic pathway are more likely to be effective. Besides targeting ergosterol biosynthetic pathway, some antifungal products are proved to have the power to penetrate and disrupt the fungal cell membranes, rich in unsaturated fatty acids, which leads to rearrangement of membrane constituents, loss of cell viability and, eventually, cell dead [31]. Here we showed that extract of *O. spinosa* provoked leakage of intracellular contents from *C. albicans* cells, which is one of the indicators pointing to cell membrane as additional target of the tested extract. Our results further showed that the extract of *O. spinosa* is complex mixture of the compounds that acted by different mechanisms. It is interesting to point that the chance of developing fungal resistance to the extract is very unlikely since the extract is acting by different mechanisms affecting different targets. It makes *O. spinosa* extract a strong candidate for future application.

### 2.5. Evaluation of Cytotoxicity of the O. spinosa Methanolic Extract on Primary Human Gingival Fibroblast Cells

Currently, a wide range of different immortalized and primary cells and tissue models are available for in vitro toxicity evaluation. The evaluation of drug cytotoxicity is an important step in biomedical research and represents a primary consideration covering drug selection. Additionally, the first step in the development of novel antifungal drugs includes toxicity studies on human cells in culture. We used human gingival fibroblast (HGF-1) cells to test possible cytotoxic effect of the extract on primary human cells.

**Figure 4:**
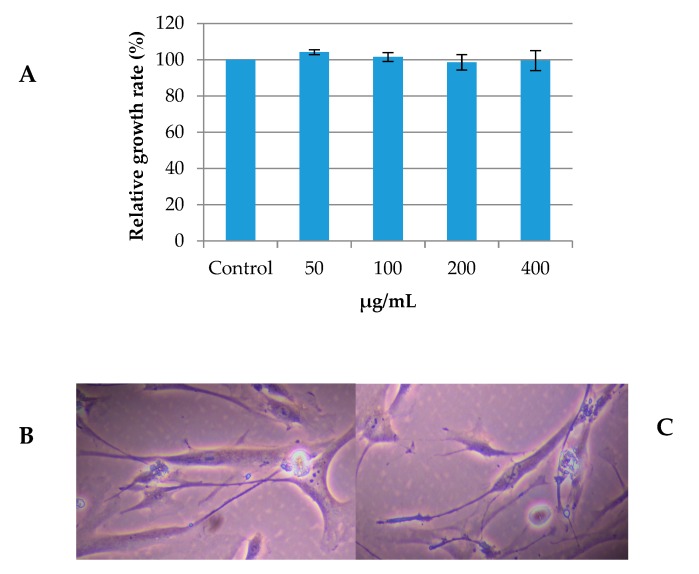
(**A**) Relative growth rate (%) of human gingival fibroblast (HGF-1) cells treated with different concentrations of *O. spinosa* extract showing no statistical difference between treatments (*p* < 0.05); (**B**) representative phase contrast image of non-treated control cells and (**C**) representative phase contrast image of cells treated with *O. spinosa* extract (400 µg/mL).

No cytotoxicity of the *O. spinosa* methanolic extract on the HGF-1 cells was observed with concentration up to 400 µg/mL; a concentrations which is considered as the limit of toxicity (Figure 4A). Namely, as shown in the Figure 4A, there was no statistical difference (*p* < 0.05) in relative growth rate between HGF-1 cells treated with different concentrations of the *O. spinosa* extract and non-treated control cells. Additionally, we analyzed morphology of the control HGF-1 cells and HGF-1 cells treated with 400 µg/mL of *O. spinosa* extract (Figure 4B,C). Obtained results revealed that the treatment of the cells with the extract did not induce changes of the cell morphology (Figure 4C) when compared to the morphology of non-treated fibroblast cells (Figure 4B). Therefore, we showed that *O. spinosa* had no influence on primary human gingival fibroblast cells, regarding the growth rate and cellular morphology.

## 3. Materials and Methods

### 3.1. Standards and Reagents

Acetonitrile (99.9%) was of high-performance liquid chromatography (HPLC) grade from Fisher Scientific (Lisbon, Portugal). Phenolic compound standards (caffeic acid, ferulic acid, hesperetin, naringenin, quercetin-3-*O*-glucoside and quercetin-3-*O*-rutinoside) were from Extrasynthèse (Genay, France). Formic acid was purchased from Sigma-Aldrich (St. Louis, MO, USA). All other general laboratory reagents were from Panreac Química S.L.U. (Barcelona, Spain). Water was treated in a Milli-Q water purification system (TGI Pure Water Systems, Greenville, SC, USA).

### 3.2. Collection and Extraction of O. spinosa

The aerial parts of wild growing *O. spinosa* were collected in Vranje (Serbia) in July 2018 and authenticated. The samples were dried, prepared and successively extracted with methanol as previously described by Ćirić et al., [32].

### 3.3. Analysis of Phenolic Compounds

The phenolic profile was determined by LC-DAD-ESI/MSn (Dionex Ultimate 3000 UPLC, Thermo Scientific, San Jose, CA, USA), and separated and identified as previously described by Bessada et al., [33]. The obtained extracts were redissolved at a concentration of 20 mg/mL with the ethanol:water (80:20, *v/v*) mixture. A double online detection was performed using a DAD (280, 330 and 370 nm as preferred wavelengths) and a mass spectrometer (MS). The MS detection was performed in a negative mode, using a Linear Ion Trap LTQ XL mass spectrometer (Thermo Finnigan, San Jose, CA, USA) equipped with an ESI source.

The identification was performed based on their chromatographic behavior and UV-vis and mass spectra by comparison with standard compounds, when available, and data reported in the literature giving a tentative identification. Data acquisition was carried out with Xcalibur^®^ data system (Thermo Finnigan, San Jose, CA, USA). In order to perform a quantitative analysis, a calibration curve for each available phenolic standard was constructed based on the UV-vis signal. The quantification of the identified phenolic compounds, for which a commercial standard was not available, was performed through the calibration curve of the most similar available standard: caffeic acid (*y* = 388345*x* + 406369, *R²* = 0.9939), ferulic acid (*y* = 633126*x* − 185462, *R²* = 0.999), hesperetin (*y* = 34156*x* + 268027, *R²* = 0.9999), naringenin (*y* = 18433*x* + 78903, *R²* = 0.9998), quercetin-3-*O*-glucoside (*y* = 34843*x* − 160173, *R²* = 0.9998), and quercetin-3-*O*-rutinoside (*y* = 13343*x* + 76751, *R²* = 0.9998). The results were expressed as mg/g of extract.

### 3.4. Antifungal Activity Assay

The antifungal activity of *O. spinosa* methanolic extract was tested against the following fungi: *Aspergillus fumigatus* (ATCC 1022), *A. niger* (ATCC 6275), *A. versicolor* (ATCC 11730), *A. ochraceus* (ATCC 12066), *P. funiculosum* (ATCC 8725), *P. ochrochloron* (ATCC 9112), *P. aurantiogriseum* (food isolate), *Trichoderma viride* (IAM 5061), *Candida albicans* (ATCC 10231), *C. tropicalis* (ATCC 750) and *C. krusei* (clinical isolate). All microorganisms used in this study were deposited at the Mycological Laboratory, Institute for Biological Research “Siniša Stanković”—National institute of Republic of Serbia, University of Belgrade, Serbia.

A modified microdilution technique was utilized to investigate the antifungal activity as described previously by Soković et al., and Clinical and Laboratory Standards Institute [34,35]. Briefly, MICs and MFCs were determined by a serial dilution technique using 96-well microtiter plates. The extract of *O. spinosa* was dissolved in 5% dimethyl-sulfoxide—DMSO. The commercial fungicides bifonazole and ketokonazole (Srbolek, Belgrade, Serbia) were used as positive controls (1–3500 μg/mL of DMSO), while 5% dimethyl-sulfoxide in water was used as a negative control.

### 3.5. Biofilm Inhibition Assay on Candida Strains

The effect of *O. spinosa* methanolic extract on biofilm formation of *C. albicans*, *C. krusei* and *C. tropicalis* was determined as previously described by Popovic et al. [36]. The extract of *O. spinosa* was dissolved in 5% dimethyl-sulfoxide—DMSO. Staining process with crystal violet was used for determination of biofilm reduction and further measuring the UV absorbance of stain at 570 nm using a plate reader. MIC was defined as the minimum concentration of antifungal agent that inhibited further growth of the initial biofilm, and minimum fungicidal concentration (MFC) was defined as the concentration presenting no fungal growth (empty well). Fluconazole (dissolved in 5% DMSO) was used as a positive control, while 5% DMSO in water was used as a negative control.

### 3.6. Insights into the mode of Antifungal Action: Ergosterol Binding and Membrane Permeability Assays

Assays were performed on the *Candida albicans* strain. Serial dilutions of the extracts were done in microtiter plates as for microdilution method with addition of ergosterol (25–100 µg/mL) [3]. After 24 h of incubation at 37 °C, MFCs were determined as explained for antifungal activity assay.

The effect of the *O. spinosa* extract on membrane permeability was evaluated as previously described by Stojković et al. [37]. Strain was incubated with the *O. spinosa* extract at the 2 × MIC for different time periods: 15, 30, 45, 60 and 90 min. *C. albicans* incubated with 10 mM PBS (pH 7.4) was used as a control. The optical density was measured at 260 nm and 280 nm (Aglient 8453 spectrophotometer) at room temperature (25 °C).

### 3.7. Investigation of O. spinosa Methanolic Extract Cytotoxic Activity

Cytotoxic effect of *O. spinosa* methanolic extract was determined on human gingival fibroblasts cells HGF-1 (ATCC^®^ CRL-2014™) using crystal violet assay as described by Feoktistova et al., [38], with some modifications. The extract of *O. spinosa* was dissolved in PBS to a final concentration of 8 mg/mL. HGF-1 cells were grown in fibroblast basal medium (ATCC^®^ PCS-201-030^TM^) at 37 °C in a CO_2_ incubator. Forty-eight hours before treatment, HGF-1 cells were seeded in a 96-well microtiter adhesive plate at a seeding density of 4 × 10^3^ cells per well. After 48 h, the medium was removed and the cells were treated for the next 48 h with various concentrations of the extract in triplicate wells. Subsequently, the medium was removed; the cells were washed twice with PBS and stained with 0.4% crystal violet staining solution for 20 min at room temperature. Afterwards, crystal violet staining solution was removed; the cells were washed in a stream of tap water and left to air dry at room temperature. The absorbance of dye dissolved in methanol was measured in a plate reader at 570 nm. The results were expressed as relative growth rate (%).

### 3.8. Statistical Analysis

All analyses were performed in triplicate; each replicate was quantified also three times. Data were expressed as mean standard deviation, where applicable. In the cases where statistical significance differences were identified, the dependent variables were compared using Tukey’s honestly significant difference (HSD) test.

## 4. Conclusions

The present study revealed underestimated biological potential of the aerial parts of *O. spinosa* plant. Methanolic extract was a good source of phenolic compounds indicated by the presence of phenolic acids, flavonoids and isoflavonoids. Flavonoids were the most dominant class of the identified compounds, followed by phenolic acids and isoflavonoids. For the first time, we reported the presence of phenolic acids in the methanolic extract of *O. spinosa*, together with the types of identified flavonoids, which were not previously reported in the investigated species. This is the first study reporting antifungal and antibiofilm activities of the methanolic extract obtained from the herb of *O. spinosa*. Based on the results it could be concluded that the extract exerted its antifungal activity by disruption of ergosterol biosynthesis and by modulation of cell membrane permeability. Finally, extract was not toxic to HGF-1 cells, which makes it the good candidate for further antifungal drug development.

## Figures and Tables

**Table 1:** Retention time (Rt), wavelengths of maximum absorption in the visible region (λ_max_), mass spectral data and tentative identification of the phenolic compounds present in *Ononis spinosa* L.

Peak	Rt (min)	λ_max ((nm))_	Molecular Ion [M − H]^−^ (*m*/*z*)	MS^2^ (*m*/*z*)	Tentative Identification	Quantification (mg/g Extract)
1	5.31	310	341	179(100),135(5)	Caffeic acid hexoside^A^	tr
2	6.55	287	355	193(100),179(5),149(5)	Ferulic acid hexoside^B^	0.078 ± 0.001
3	9.93	310	179	135(100)	Caffeic acid^A^	0.020 ± 0.001
4	13.66	328	473	311(100),293(92),267(5),179(5),149(8),135(5)	*cis* Chicoric acid ^A^	0.81 ± 0.02
5	14.17	328	473	311(100),293(90),267(5),179(5),149(10),135(5)	*trans* Chicoric acid^A^	0.76 ± 0.01
6	15.96	348	595	301(100)	Quercetin-*O*-hexoside-pentoside^C^	0.824 ± 0.001
7	16.43	341	609	285(100)	Kaempherol-*O*-dihexoside^C^	0.732 ± 0.002
8	17.11	305	459	297(100)	Spinonin-*O*-hexoside^E^	tr
9	18.5	328	463	301(100)	Quercetin-3-*O*-glucoside^D^	0.61 ± 0.01
10	19.07	346	579	285(100)	Kaempherol-*O*-hexoside-pentoside^C^	5.1 ± 0.2
11	20.58	348	505	463(70),301(100)	Acetylquercetin-*O*-hexoside^D^	0.566 ± 0.002
12	21.18	337	447	285(100)	Kaempherol-*O*-hexoside^D^	0.58 ± 0.01
13	22.37	340	447	285(100)	Kaempherol-3-*O*-glucoside^D^	0.71 ± 0.02
14	25.34	262/291	489	281(100)	Pseudobaptigenin-*O*-hexoside^E^	0.134 ± 0.003
15	26.56	261/310	475	267(100)	Formononetin derivative^F^	1.28 ± 0.02
16	31.18	260/310	515	471(15),429(5),267(100)	Formononetin-*O*-malonyl-hexoside^F^	tr
					**Total phenolic acids**	**1.66 ± 0.03**
					**Total isoflavonoids**	**1.41 ± 0.02**
					**Total flavonoids**	**9.1 ± 0.1**
					**Total phenolic compounds**	**12.2 ± 0.1**

Tr—traces. Standard calibration curves: ^A^—caffeic acid (*y* = 388345*x* + 406369, *R²* = 0.9939), ^B^—ferulic acid (*y* = 633126*x* − 185462, *R²* = 0.999), ^C^—quercetin-3-*O*-rutinoside (*y* = 13343*x* + 76751, *R²* = 0.9998), ^D^—quercetin-3-*O*-glucoside (*y* = 34843*x* − 160173, *R²* = 0.9998), ^E^—naringenin (*y* = 18433*x* + 78903, *R²* = 0.9998), ^F^—hesperetin (*y* = 34156*x* + 268027, *R²* = 0.9999).

**Table 2:** Antifungal activities of *O. spinosa* methanolic extract (MIC (minimum inhibitory concentration) and MFC (minimum fungicidal concentration) mg/mL).

Microfungi		*O. spinosa*	Ketoconazole	Bifonazole
*A. fumigatus* (ATCC 9197)	MIC	0.08 ^b^	0.20 ^a^	0.15 ^b^
	MFC	0.08 ^b^	0.50 ^b^	0.20 ^a^
*A. versicolor* (ATCC 11730)	MIC	0.62 ^d^	0.20 ^a^	0.10 ^a^
	MFC	1.25 ^d^	0.50 ^b^	0.20 ^a^
*A. niger* (ATCC 6275)	MIC	0.62 ^d^	0.20 ^a^	0.15 ^b^
	MFC	1.25 ^d^	0.50 ^b^	0.20 ^a^
*A. ochraceus* (ATCC 12066)	MIC	2.50 ^e^	1.50 ^e^	0.15 ^b^
	MFC	2.50 ^e^	2.00 ^e^	0.20 ^a^
*Trichoderma viride* (IAM 5061)	MIC	0.62 ^d^	1.00 ^d^	0.15 ^b^
	MFC	1.25 ^d^	1.00 ^c^	0.20 ^a^
*Penicillium funiculosum* (ATCC 36839)	MIC	0.62 ^d^	0.20 ^a^	0.20 ^c^
	MFC	1.25 ^d^	0.50 ^b^	0.25 ^b^
*P. aurantiogriseum* (food isolate)	MIC	0.02 ^a^	0.20 ^a^	0.10 ^a^
	MFC	0.04 ^a^	0.30 ^a^	0.20 ^a^
*P. ochrochloron* (ATCC 9122)	MIC	5.00 ^f^	1.00 ^d^	0.20 ^c^
	MFC	10.00 ^f^	1.50 ^d^	0.25 ^b^
*Candida albicans* (ATCC 10231)	MIC	0.62 ^d^	0.50 ^c^	0.15 ^b^
	MFC	1.25 ^d^	1.00 ^c^	0.30 ^c^
*C. krusei* (clinical isolate)	MIC	0.62 ^d^	0.50 ^c^	0.25 ^d^
	MFC	1.25 ^d^	1.00 ^c^	0.50 ^d^
*C. tropicalis* (ATCC 750)	MIC	0.31 ^c^	0.30 ^b^	0.25 ^d^
	MFC	0.62 ^c^	0.50 ^b^	0.50 ^d^

In each column different letters means significant difference between MICs and MFCs values for each fungal species tested (*p* < 0.05).

**Table 3:** Activity of *O. spinosa* extract and a reference compound fluconazole against biofilm (plaque) formation of *Candida* strains (MIC and MFC, mg/mL).

Fungi	*O. spinosa* Methanolic Extract		Fluconazole	
	MIC	MFC	MIC	MFC
*C. albicans* (ATCC 10231)	5.00 ^b^	10.00 ^b^	8.00 ^c^	9.00 ^c^
*C. krusei* (clinical isolate)	2.50 ^a^	5.00 ^a^	2.00 ^a^	3.00 ^a^
*C. tropicalis* (ATCC 750)	5.00 ^b^	10.00 ^b^	3.00 ^b^	6.00 ^b^

In each column different letters means significant difference between MICs and MFCs values for each fungal species tested (*p* < 0.05).
